# Supplementary figures and images for: Combinations of Lemongrass and Star Anise Essential Oils and Their Main Constituent: Synergistic Housefly Repellency and Safety against Non-Target Organisms
Source: Insects. 2024 Mar 20;15(3):210. doi: 10.3390/insects15030210 (PMC10971264; doi:10.3390/insects15030210)

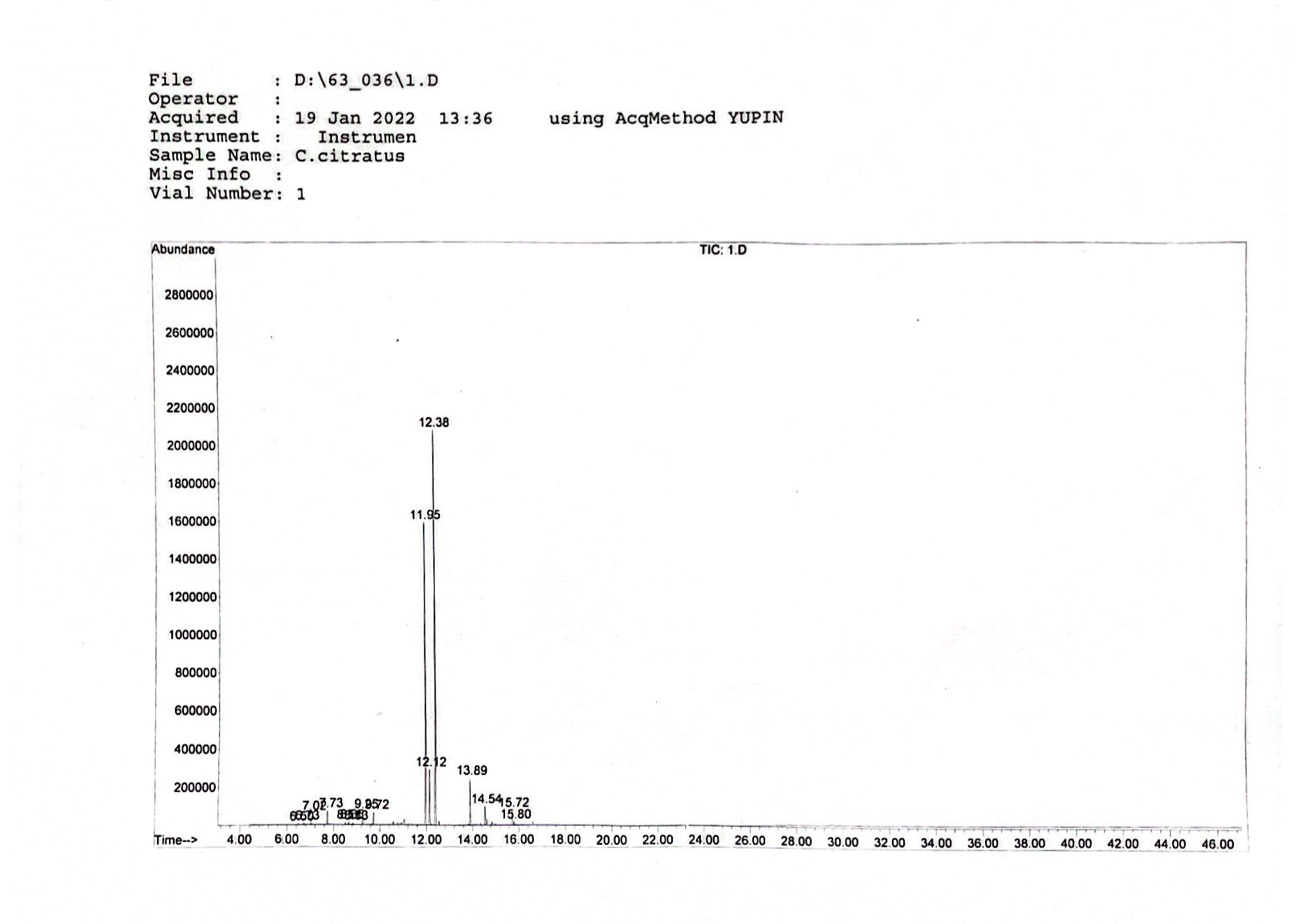

Supplement: Supplementary file 1 [file insects-15-00210-s001.zip › Figure S1-GC-MS lemongrass.png]

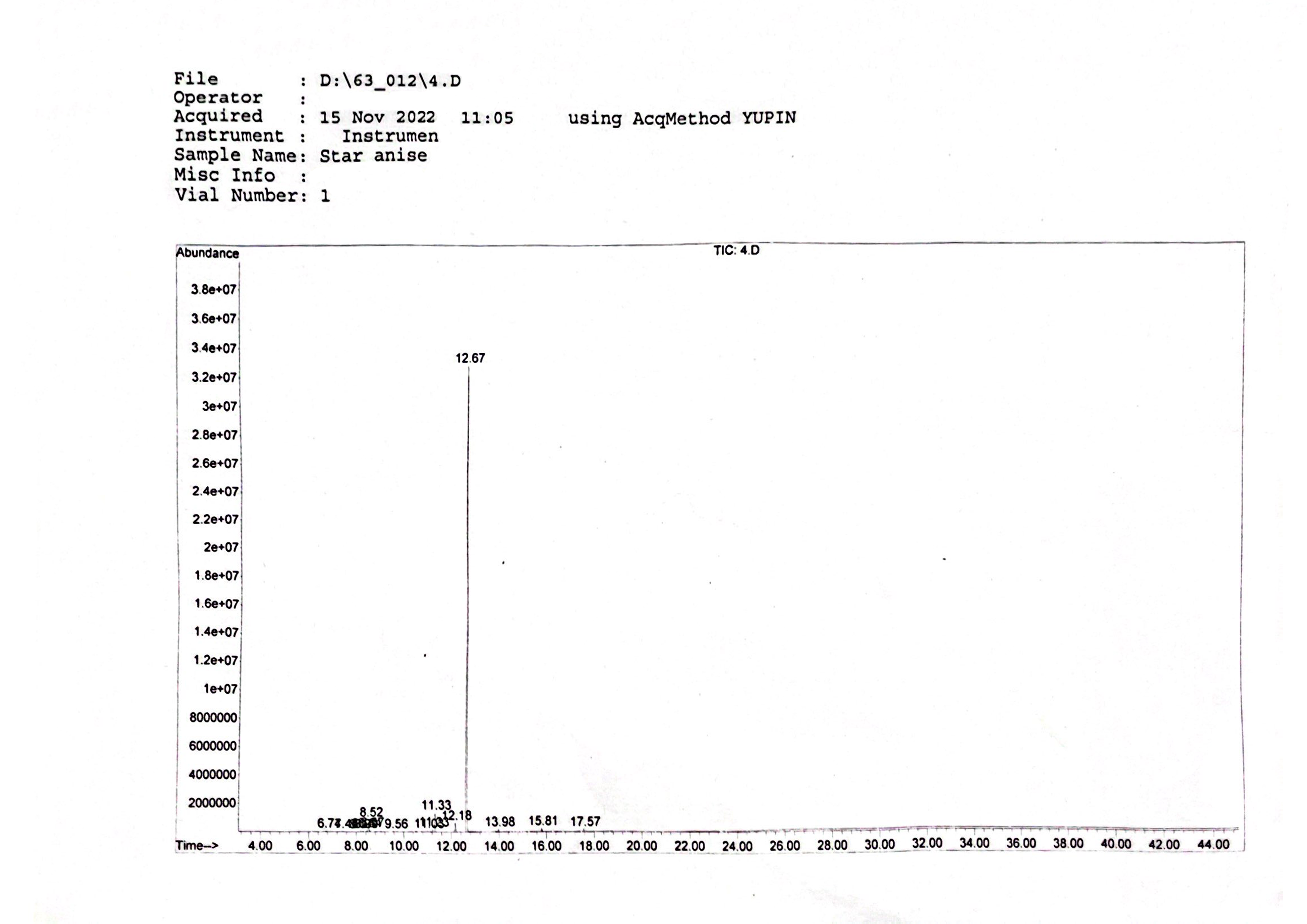

Supplement: Supplementary file 1 [file insects-15-00210-s001.zip › Figure S2-GC-MS star anise.png]
